# Peripheral PD-1+CD56+ T-cell frequencies correlate with outcome in stage IV melanoma under PD-1 blockade

**DOI:** 10.1371/journal.pone.0221301

**Published:** 2019-08-16

**Authors:** Jonas Bochem, Henning Zelba, Teresa Amaral, Janine Spreuer, Daniel Soffel, Thomas Eigentler, Nikolaus Benjamin Wagner, Ugur Uslu, Patrick Terheyden, Friedegund Meier, Claus Garbe, Graham Pawelec, Benjamin Weide, Kilian Wistuba-Hamprecht

**Affiliations:** 1 Department of Dermatology, University Medical Center, Tübingen, Germany; 2 Portuguese Air Force Health Direction, Lisbon, Portugal; 3 Department of Dermatology, Friedrich-Alexander-University of Erlangen-Nürnberg (FAU), Universitätsklinikum Erlangen, Erlangen, Germany; 4 Department of Dermatology, University of Lübeck, Lübeck, Germany; 5 Skin Cancer Center at the University Cancer Centre, Department of Dermatology, Faculty of Medicine and University Hospital Carl Gustav Carus, Technische Universität Dresden, Dresden, Germany; 6 National Center for Tumor Diseases (NCT), Dresden, and German Cancer Research Center (DKFZ), Heidelberg, Germany; 7 Department of Immunology, University of Tübingen, Tübingen, Germany; 8 Health Sciences North Research Institute, Sudbury, Ontario, Canada; 9 Division of Cancer Studies, King’s College London, London, United Kingdom; 10 John van Geest Cancer Research Centre, Nottingham Trent University, Nottingham, United Kingdom; 11 Institute of Cancer Sciences, Manchester University, Manchester, United Kingdom; Université Paris Descartes, FRANCE

## Abstract

Immune checkpoint blockade with anti-PD-1 antibodies is showing great promise for patients with metastatic melanoma and other malignancies, but despite good responses by some patients who achieve partial or complete regression, many others still do not respond. Here, we sought peripheral blood T-cell biomarker candidates predicting treatment outcome in 75 stage IV melanoma patients treated with anti-PD-1 antibodies. We investigated associations with clinical response, progression-free survival (PFS) and overall survival (OS). Univariate analysis of potential biological confounders and known biomarkers, and a multivariate model, was used to determine statistical independence of associations between candidate biomarkers and clinical outcomes. We found that a lower than median frequency of peripheral PD-1+CD56+ T-cells was associated with longer OS (p = 0.004), PFS (p = 0.041) and superior clinical benefit (p = 0.009). However, neither frequencies of CD56-CD4+ nor CD56-CD8+ T-cells, nor of the PD-1+ fraction within the CD4 or CD8 subsets was associated with clinical outcome. In a multivariate model with known confounders and biomarkers only the M-category (HR, 3.11; p = 0.007) and the frequency of PD-1+CD56+ T-cells (HR, 2.39; p = 0.028) were identified as independent predictive factors for clinical outcome under PD-1 blockade. Thus, a lower than median frequency of peripheral blood PD-1+CD56+ T-cells prior to starting anti-PD-1 checkpoint blockade is associated with superior clinical response, longer PFS and OS of stage IV melanoma patients.

## Introduction

Recent innovations in cancer treatment have led to great success in late-stage melanoma[1,2]. The first FDA/EMA-approved checkpoint inhibitor, the monoclonal antibody ipilimumab against cytotoxic T-lymphocyte–associated antigen 4 (CTLA-4) expressed on the surface of T-cells, and antagonistic antibodies targeting programmed death-1 (PD-1), such as nivolumab and pembrolizumab, yielded an improvement in response rates, progression-free survival (PFS) and overall survival (OS) in patients with advanced melanoma[3–5]. Although a proportion of patients responds to these agents and benefits from long-lasting remissions, there are many non-responding patients who may nonetheless suffer side effects[6]. Therefore, the search for biomarkers indicative of a response to a certain treatment and predicting the outcome and potential associated toxicity is of importance. The source of material for such assays ideally needs to be easy and fast to access, guaranteeing later clinical workability. Peripheral blood fulfills these requirements and is currently the source of the only validated biomarker in late-stage melanoma in routine use, namely serum lactate dehydrogenase (LDH) levels[7]. Recent studies revealed several potential biomarker candidates associating with outcome of immune checkpoint therapy in metastatic melanoma, such as the frequencies of peripheral blood myeloid-derived suppressor cells (MDSCs) and defined T-cell subsets in patients treated with ipilimumab[8–10]. More recently in patients treated with anti-PD-1 antibodies, frequencies of classical monocytes in the periphery[11] or a reinvigoration of circulating exhausted, Ki65+PD-1+CD8+ T-cells in conjunction with tumor burden were recently suggested as measures to predict poor clinical responses[12].

In addition to the well-documented CD4+ and CD8+ T-cell subsets, another peripheral subset of T-cells, expressing the neural cell adhesion molecule 1 (NCAM1; CD56), commonly expressed on group I innate lymphoid NK-cells[13,14], has been relatively poorly investigated in the context of melanoma immunotherapy. This CD56+ T-cell population represents a phenotypically and functionally heterogeneous population, divided into type I[15,16] and type II[17,18] Natural Killer T (NKT) cells. Further, CD56 expression on CD8+ and CD4-CD8- T-cells can be induced by TCR-mediated activation, resulting in poor proliferative capacity but enhanced cytotoxicity[19]. However, additional T-cell populations, such as γδ T-cells[20,21] and regulatory T-cells (Tregs)[22] can also express CD56. In cancer, higher frequencies of this heterogeneous population and functional impairments thereof relative to healthy controls have been reported[23–25]. However, there is also evidence that peripheral CD56+ T-cell frequencies might be affected by other diseases such as psoriasis[26], vitiligo[27] and viral infections like human cytomegalovirus (CMV)[28] and chronic Hepatitis B[29].

The aim of the present study was to investigate peripheral CD56+ T-cell populations and the therapeutically relevant PD-1+ fraction thereof in stage IV melanoma before the initiation of PD-1 immune checkpoint therapy. Correlations with clinical meta-data such as serum LDH-levels, M-category, response evaluation according to Response Evaluation Criteria in Solid Tumors (RECIST) version 1.1[30], PFS and OS were performed to evaluate this T-cell subpopulation as a novel biomarker-candidate for outcome of PD-1 immune blockade.

## Materials and methods

### Patients

Patients’ blood samples were obtained between March 2015 and March 2017 from three different clinical centers: Tübingen, Dresden, Lübeck. Sample size calculation using nQuery Advisor revealed that at least 68 patients were required to identify a biomarker with clinical relevance (improvement in one-year OS), when the observed cohort is dichotomized by the latter in two balanced groups (equal n). Clinical relevance was defined as 80% one-year OS rate in the favorable and 60% in the unfavorable group considering α = 0.05 and a power of 80% (1-sided). The total sample size for this study was set to 75 patients, assuming a drop-out rate of 10%. All 75 included patients had stage IV melanoma with distant metastases, received no previous PD-1 immune checkpoint blockade and donated blood just before starting therapy with anti-PD-1 antibodies. Patients received either 2-3mg/kg Pembrolizumab every 3 weeks or 3mg/kg Nivolumab every 2 weeks. Within 24 hours of blood draw, peripheral blood mononuclear cells (PBMCs) were centrally isolated in Tübingen using Ficoll-Hypaque density gradient centrifugation and were immediately cryopreserved. Anti CMV-specific antibody screening was performed using Cobas 6000 / Cobas e 601 analyzer with quantitative Elecsys CMV IgG (U/ml) assays (Roche diagnostics). Relevant clinical metadata were documented for each patient. All patients gave written informed consent for biobanking and use of biomaterial and clinical data for scientific purposes. This study and experimental procedures were approved by the local Tübingen Ethics Committee (490/2014BO1 and 792/2016BO2). The anonymized raw dataset is summarized in S1 Table.

### Flow cytometry

Cryopreserved PBMCs were thawed and 1x10^6^ cells per sample washed with PBS containing 2% FCS, 2 mM EDTA, and 0.01% sodium azide at room temperature. After Fc receptor blocking with Gamunex (human immunoglobulin; GRIFOLS) and labeling of dead cells with ethidium monoazide (EMA; Biotinum), the cells were stained with the following titrated monoclonal antibodies: CD3-A700 (clone UCHT1, Biolegend), CD56-FITC (clone HCD56, Biolegend), CD4-PerCP (clone SK3, BD Bioscience), CD8-APC-Cy7 (clone SK1, Biolegend) and PD-1-BV711 (clone EH12.2H7, Biolegend). Lastly, after additional washing the samples were measured with a LSRII cytometer (BD) and processed with BD FACSDiva software 6.1.3. Single color controls were used for automatically calculated compensation.

### Flow cytometry data analysis

The analysis of the resulting flow cytometry data was performed using Flowjo 10.4 (Treestar). An example of the applied gating strategy is displayed in S1 Fig. To monitor potential inter-batch discrepancies, one sample deriving from the same large standard batch of PBMCs from a healthy subject was included in each patient sample run (day of measurement). After setting a time gate to identify potential fluctuations in the flow rate and an FSC-A versus FCC-H and an SSC-A versus SSC-H gate to exclude debris, duplicates and dead cells were excluded (i.e. ethidium monoazide bromide [EMA]-positive events). Within viable and morphologically-gated lymphocytes (SSC-A versus FSC-A) the CD56+CD3+ and CD56-CD3+ T-cells were selected and further subdivided into CD8+, CD4+ and double-negative (DN) T-cells. All populations were characterized for PD-1 expression. A threshold of minimally 120 processed events was set as cut-off population size for subdivision in daughter populations.

### Statistical data analysis

Throughout this work, ≤median frequency of the cell population of interest was used as cutoff to dichotomize the cohort for survival correlations. OS was defined from the first dose of antagonistic PD-1 antibodies to either the death of a patient due to disease or to the date of last contact. Deaths for other reasons were considered as censored events. Survival probabilities were estimated by the Kaplan-Meier approach and compared by log-rank testing. PFS was defined as the time from the date of starting therapy to the date of progression or death. Elevated/normal serum LDH was determined by the actual value in relation to the upper limit of normal. A stepwise Cox regression analysis with backward variable selection based on p-values was performed to determine the relative impact of single predictive features. Results of the Cox regression analysis are described by means of hazard ratios (HR), and p-values (Wald test). Patients with missing values for at least one feature were excluded.

Response to therapy was evaluated by local physicians at the respective centers following the Response Evaluation Criteria in Solid Tumors (RECIST) version 1.1[30] and was categorized as complete response (CR), partial response (PR), stable disease (SD) or progressive disease (PD). We defined clinical response to therapy as best overall response between the initiation of therapy and progression, or start of a new systemic therapy, considering all available tumor assessments within this period of time. Clinical benefit analysis included patients experiencing CR, PR and SD contrasted with patients suffering PD. Fisher’s exact test (two-sided) was used to evaluate the significance of differences of these correlations.

T-distributed stochastic neighbor embedding (tSNE) analysis was used to visualize the composition of the heterogeneous CD56+ T-cell subset and its PD-1 expression pattern using FlowJo and the embedded tSNE approach[31]. Prior to application of the latter, all CD56+ T-cells of each patient were extracted from the raw data files (fcs-format), down-sampled (using the respective tool in the FlowJo software) and transferred into new fcs-files, that were used for downstream analysis. Expression of CD4 and CD8 was the basis for the clustering. The same gating strategy as described above was used to visualize the presence/absence of CD4, CD8 and PD-1 on the respective tSNE plot.

The Mann-Whitney U test was used for group-wise comparisons, while the Kruskal-Wallis test was used for statistical evaluation of groups with n>2. P values <0.05 were considered significant throughout the study. SPSS 24 and Prism 6 (GraphPad) were used for statistical calculations.

## Results

### Patients and treatments

Cryopreserved PBMCs from 75 stage IV melanoma patients prior to infusion of antagonistic PD-1 antibodies (pembrolizumab (n = 70) or nivolumab (n = 5)) were provided by three clinical centers (Table 1). Of these 75 patients, 45 were male (60%), 53 were CMV-IgG seropositive (70.7%) and the median age was 73 years (interquartile-range 62 to 79 years). Clinical response was assessed following the Response Evaluation Criteria in Solid Tumors (RECIST) version 1.1[30]. Best overall response was defined as best clinical response between the initiation of PD-1 immune checkpoint therapy and progression, or start of a new systemic treatment: Twelve patients experienced CR (16%), 9 PR (12%), 12 SD (16%) and 42 patients had PD (56%). The M-category was defined according to the American Joint Committee on Cancer (AJCC) recommendations[32]. Fifteen patients were classified as M1a (20%), 19 patients were M1b (25.3%) and 35 patients M1c (46.7%). Six patients could not be classified. Details are summarized in Table 1. Median survival of the cohort was not reached, the median PFS was 128 days and the median follow up was nearly two years (709 days).

**Table 1:** Cohort characteristics.

Variable	Category	Patients n	Patients %
Sex	Male	45	60
	Female	30	40
Age	≤60	17	22.7
	>60	16	21.3
	>70	25	33.3
	>80	17	22.7
	Median age	73 years	
Treatment	Pembrolizumab	70	93.3
	Nivolumab	5	6.7
Center	Tübingen	55	73.3
	Dresden	13	17.3
	Lübeck	7	9.3
M-category	M1a	15	20
	M1b	19	25.3
	M1c	35	46.7
	unknown	6	8
CMV serostatus	Seropositive	53	70.7
	Seronegative	21	28
	unknown	1	1.3
Serum LDH	normal	51	68
	elevated	24	32
Best clinical response (RECIST)	CR	12	16
	PR	9	12
	SD	12	16
	PD	42	56

### T-cell profiling

**Fig 1:**
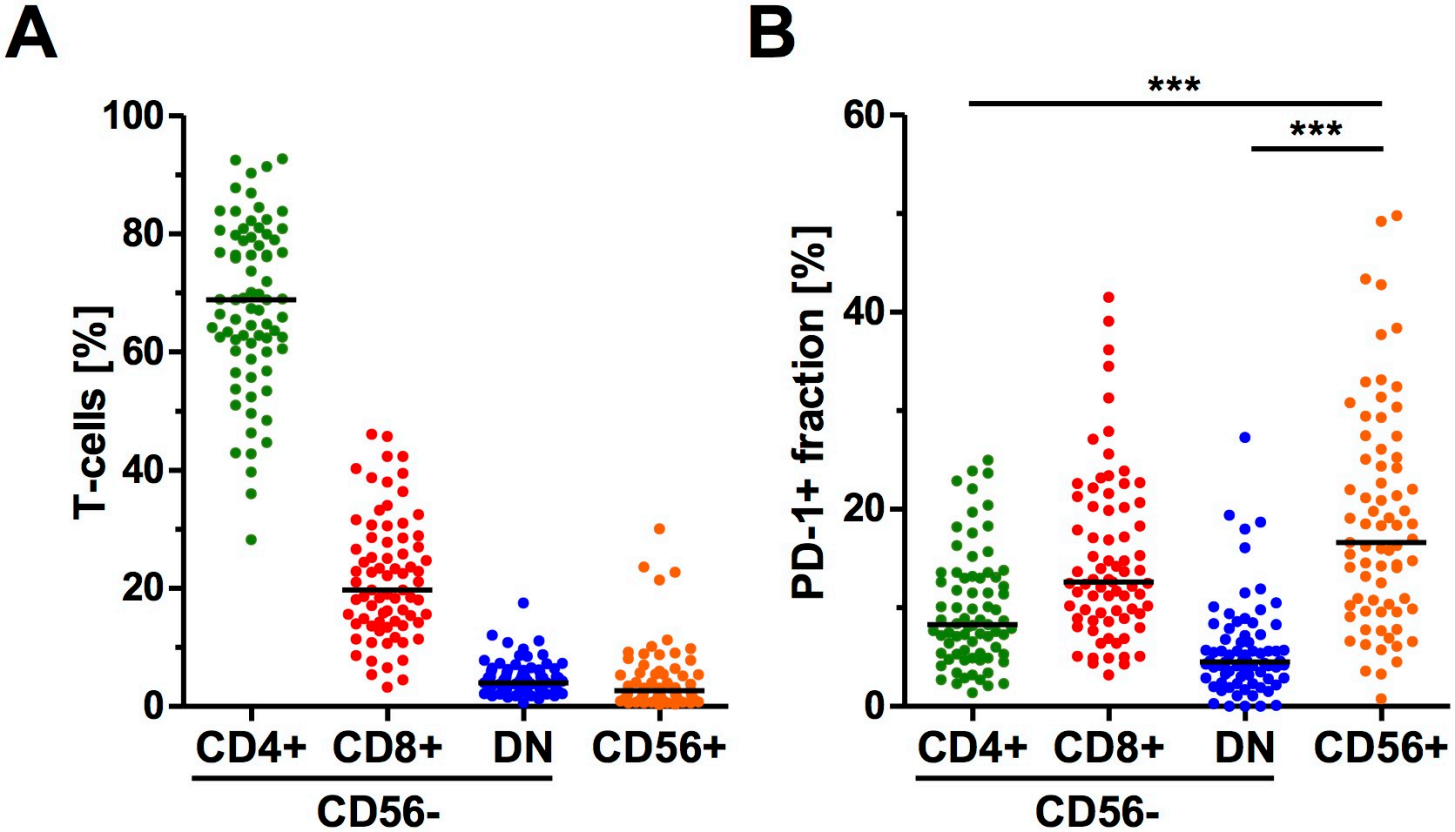
Peripheral T-cell profile. Frequencies of CD56- CD4+, CD8+, DN and CD56+ T-cell subsets (n = 75 for all) in the CD3+ T-cell population (A) and the PD-1+ fraction within these subsets (B) in melanoma patients prior to initiation of immune therapy using antagonistic PD-1 antibodies. Two samples could not be analyzed for the PD-1+ fraction within the CD56+ T-cells. Lines in the dot plots indicate the population median and each dot represents an individual patient; *** p < 0.001 using the Kruskal-Wallis test.

The peripheral CD3+ T-cell compartment was divided into the following major phenotypes: CD56-CD4+, CD56-CD8+, CD56-CD4-CD8-(DN) and CD56+ (S1 Fig). The largest peripheral T-cell phenotype according to frequency was CD56-CD4+ with a median of 68.8% of all CD3+ T-cells. The second most common was CD56-CD8+ with 19.7% in median, whereas CD56-DN cells were present at a median frequency of only 4.1%. An even smaller proportion of all CD3+ T-cells was CD56+ (median 2.7%, Fig 1A). However, this CD56+ T-cell population contained the largest proportion of PD-1+ cells (median frequency of 16.6%) relative to all the other T-cell populations (i.e. medians of 12.7% of CD56-CD8+, 8.3% of CD56-CD4+ and 4.5% of CD56-DN T-cell subset, Fig 1B). Of note, frequencies of the PD-1+ T-cells did not correlate with frequencies of the parental T-cell populations.

All patients were serotyped to determine anti-CMV specific IgG titers indicative of a latent infection with CMV. The latter is known to have a profound impact on the distribution of peripheral T-cell phenotypes. For this reason, it is included here as a potential confounding factor for the analysis of tumor-associated immune cell marker patterns[28,33]. In accordance with previous reports in healthy controls, CMV-seropositive patients possessed significantly higher frequencies of CD8+ T cells (23.4% versus 17.4% median; p = 0.033), lower frequencies of CD4+ T cells (69.7% versus 77% median; p = 0.005) and a greater abundance of CD56+ T cells (3.1% versus 1.2% median; p≤ 0.001) relative to CMV-seronegative patients (S2A Fig)[28,33]. However, CMV-seropositivity had no significant impact on the abundance of any of the PD-1+ T-cell subsets analyzed here (S2B Fig).

### Correlation of T-cell subsets with survival and response

**Fig 2:**
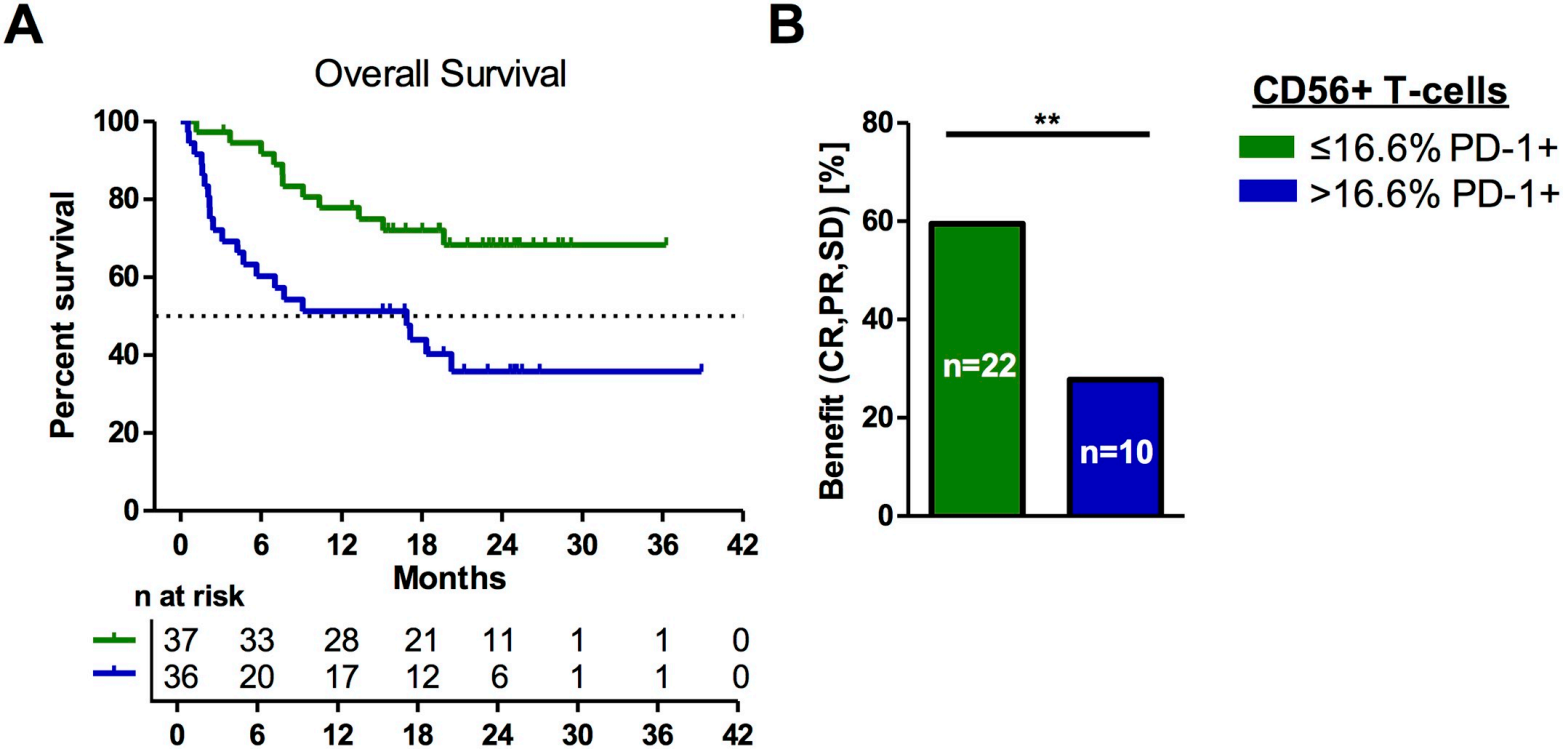
Peripheral PD-1+CD56+ T-cell frequencies correlate with clinical outcome after therapy. Probability of overall survival among patients with >16.6% [blue] and ≤16.6% [green] peripheral PD-1+CD56+ T-cell frequencies prior to the start of therapy were analyzed using the Kaplan Meier approach (p = 0.004; log-rank test) (A). Vertical lines indicate censored events. The number of patients that experienced a clinical benefit from therapy (complete responder, partial responder or stable disease) with either >16.6% (blue) or ≤16.6% (green) PD-1+CD56+ T-cell frequencies are displayed in (B) (** p≤ 0.01; Fisher’s exact test, two-sided).

Correlations of OS with frequencies of the major peripheral blood phenotypes CD56-CD4+, CD56-CD8+, CD56-DN and CD56+ T-cells among all CD3+ T-cells did not reveal any statistically significant associations (Table 2). Next, we quantified the proportions of these 4 T-cell populations expressing PD-1. None of the observed PD-1+ T-cell phenotypes was informative for outcome after immune checkpoint therapy, except the abundance of PD-1+ T-cells within the CD56+ T-cell population. The frequency of these cells correlated negatively with OS when dichotomized by median frequency (p = 0.004; Fig 2A). Thus, patients with >16.6% of circulating PD-1+CD56+ T-cells had a 1-year OS rate of 52.8% (19 of 36), while patients in the reciprocal group (≤16.6%) had a 1-year OS rate of 78.4% (29 of 37). Patients in the group with superior OS also had a significantly longer PFS than the reciprocal group (p = 0.041; S3 Fig). The 1-year PFS-rate was only 27.8% (10 of 36) in the group of patients defined through high frequencies of PD-1+CD56+ T-cells, compared to 35.1% for those in the reciprocal group (13 of 37). Next, we investigated whether the PD-1+CD56+ T-cell population also correlated with clinical response to PD-1 immune checkpoint therapy and found that 59.5% of patients in the group with a low frequency of PD-1+CD56+ T-cells (≤16.6%) (22 of 37) experienced a benefit (CR, PR or SD) in contrast to only 27.8% patients (10 of 36) in the reciprocal group (p = 0.009) (Fig 2B).

**Table 2:** Univariate T-cell phenotype OS analysis.

Variable	Total n	Categories	n	%dead	1-year survival rate n (%)	P-value
CD4+ CD56- T-cells	75	≤68.8	39	38.5	28 (71.8)	0.245
		>68.8	36	50	22 (61.1)	
CD8+ CD56- T-cells	75	≤19.7	38	52.6	22 (57.9)	0.085
		>19.7	37	35.1	28 (75.7)	
CD4-CD8- CD56- T-cells	75	≤4.1	38	44.7	25 (65.8)	0.961
		>4.1	37	46.8	25 (67.6)	
CD56+ T-cells	75	≤2.7	40	42.5	27 (67.5)	0.677
		>2.7	35	45.7	23 (65.7)	

### Investigation of dependencies between PD-1+CD56+ T-cells and other potentially confounding features for associations with OS

**Fig 3:**
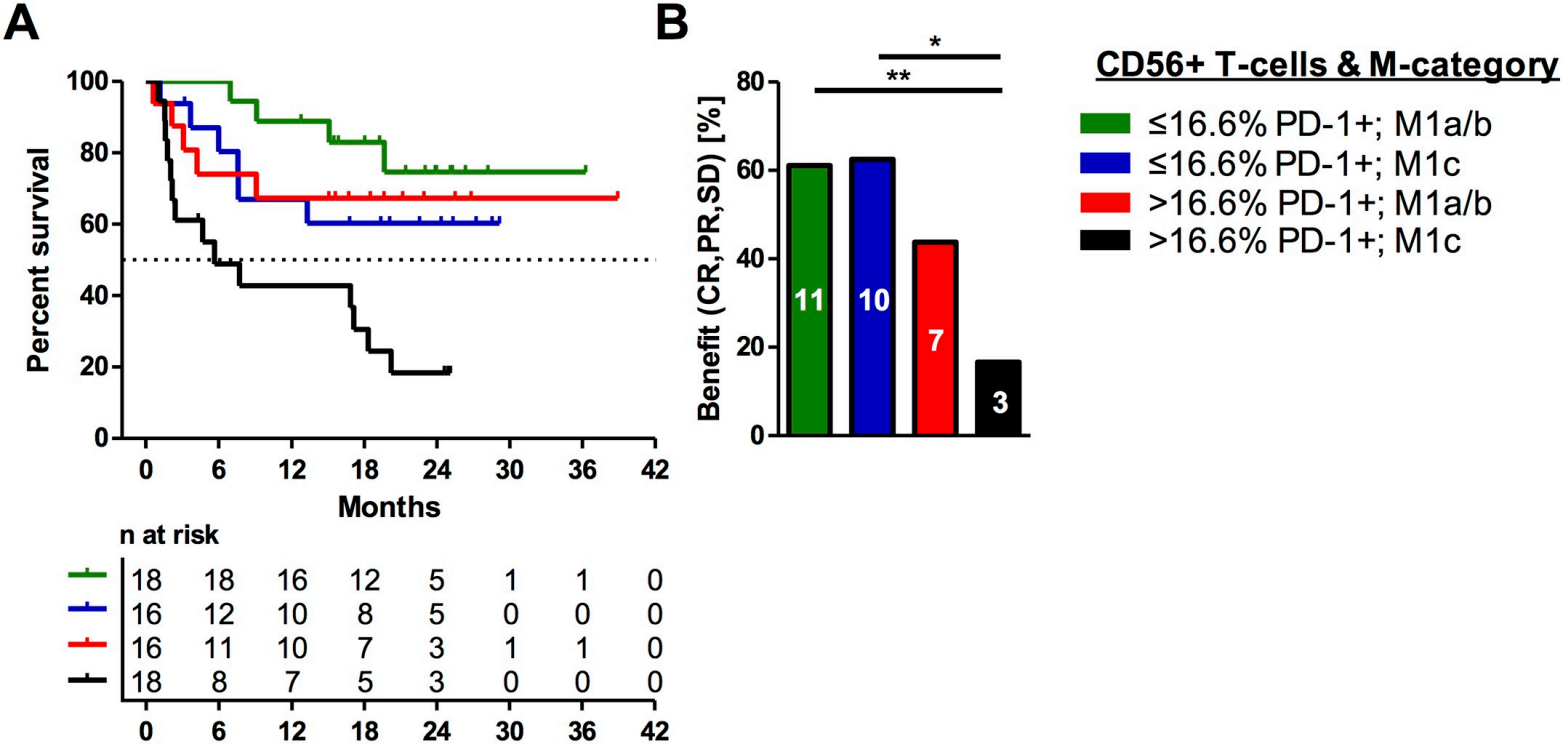
Combinatory model. The combinatory model comprising the predictive capacity of two independent predictive features: peripheral PD-1+CD56+ T-cell frequencies and the M-category. Superior survivors (green) are characterized by low abundance of PD-1+CD56+ T-cells and grouping in M1a/b. Reciprocally, patients with high frequencies of PD-1+CD56+ T-cells and grouping in the M1c category (black) had the poorest outcome (A). Vertical lines indicate censored events in the Kaplan Meier plot. Analysis of clinical benefit from PD-1 immune checkpoint therapy is shown accordantly with absolute numbers of patients with clinical benefit in these groups in (B) (* p ≤0.05; ** p ≤ 0.01). Statistical evaluation was performed by two-sided Fisher exact test.

To further investigate the predictive capacity of peripheral PD-1+CD56+ T-cell frequencies for effective PD-1 immune checkpoint therapy, we ran a descriptive, step-wise multivariate analysis that combined this variable with the M-category (M1a/b versus M1c), serum LDH (normal versus elevated); CMV-serostatus, sex, age (≤73 versus >73), PD-1+CD4+ T-cells (≤8.3% versus >8.3%), and PD-1+CD8+ T-cells (≤12.7% versus >12.7%). This analysis included 68 patients, for which all variables were available, while 7 patients were excluded because at least one parameter was missing. Interestingly, univariate analysis of these features identified only the M-category and PD-1+CD56+ T-cells as correlating with OS (p = 0.005; p = 0.004, respectively; S4 Fig). Moreover, multivariate analysis identified the PD-1+CD56+ frequencies (HR, 2.39; p = 0.028) and the M-category (HR, 3.11; p = 0.007) as independent predictive features for outcome under PD-1 immune checkpoint therapy. The combination of these two independent predictive features allows the identification of two extreme groups: very poor (M1c plus > 16.6% peripheral PD-1+CD56+ T-cells) and superior survivors (M1a/b plus ≤16.6% peripheral PD-1+CD56+ T-cells) (OS p≤ 0.001; PFS = 0.030; clinical benefit 3 of 18 versus 11 of 18 p = 0.006; Fig 3).

### Composition of the peripheral CD56+ T-cell population

**Fig 4:**
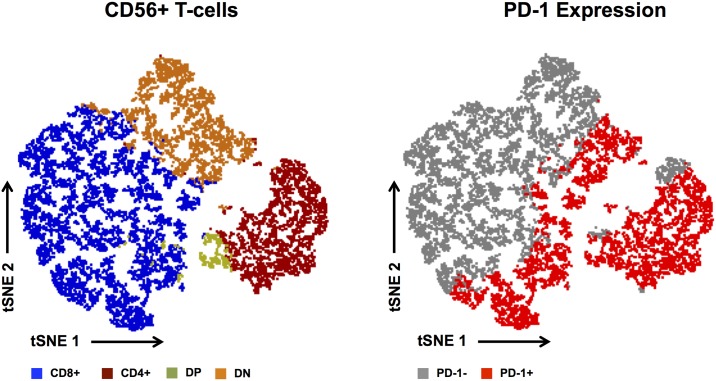
Composition and PD-1 expression of the CD56+ T-cell population. The composition of the CD56+ T-cell population is visualized in a tSNE map that comprises all samples of the observed cohort. Clustering based on CD4 and CD8 expression visualized the 4 different subsets (CD8 in blue, CD4 in red, double positive in green and double negative in orange) (A). PD-1 expression is highlighted in red (B). Each dot in the maps represents a single cell and its color the phenotype based on manual gating.

Finally, we analyzed the composition of the heterogeneous CD56+ T-cell population using the t-distributed stochastic neighbor embedding algorithm (tSNE)[31] on the entire data set (n = 73). The CD56+ T-cell population consisted of a median of 40.3% CD8+, 16.4% CD4+, 28.6% DN and 3.8% CD4+CD8+ (DP) cells (Fig 4A). The tSNE map presented in Fig 4B is utilized to visualize the distribution of PD-1 expression on all CD56+ T-cells. The latter was found on all four subsets of the CD56+ T-cell population (Fig 4B), suggesting that it is the expression of CD56 itself regardless of which type of T-cell expresses it (i.e. not the compartmentalization of T-cells by presence/absence of CD4 and/or CD8) that should be considered when searching for direct targets of PD-1 immune checkpoint therapy.

## Discussion

Earlier work has identified peripheral immune cell populations such as MDSCs or effector T-cell phenotypes as promising biomarker candidates for the clinical outcome of anti-CTLA4 checkpoint blockade in metastatic melanoma[8–10]. However, different biomarker-candidates have been recently suggested for correlations with outcome for PD-1 immune checkpoint therapy. For example, frequencies of classical monocytes[11], reinvigoration of a circulating exhausted T-cell population in conjunction with pre-treatment tumor burden[12] or a distinct activated tumor-resident effector memory T-cell population[34] were reported to be informative for outcome of PD-1 immune checkpoint therapy in late stage melanoma.

The focus of the present biomarker discovery study in stage IV melanoma was the investigation of T-cell populations in patients receiving PD-1 immune checkpoint therapy. We performed a detailed analysis of circulating T-cell subsets, including unconventional CD56+ T-cells, and their PD-1+ fraction prior to the infusion of antagonistic PD-1 antibodies. We identified a strong inverse correlation of peripheral frequencies of PD-1+CD56+ T-cells before the start of PD-1 immune checkpoint therapy with clinical benefit, PFS and OS. Moreover, this population contained the highest frequency of PD-1+ T-cells compared to all observed T-cell populations and is thereby likely to be a prominent target of the applied therapy. Thus, our data identify a promising predictive biomarker candidate for response to and outcome after anti-PD-1 immune checkpoint blockade.

PD-1 is upregulated on T-cells in response to antigen exposure and is of particular interest for the identification of cancer antigen-reactive peripheral and tumor-infiltrating CD8+ T-cell populations in melanoma[35–37]. However, we identified neither correlations between frequencies of classical circulating CD4+ or CD8+ T-cells lacking CD56 expression, nor their PD-1+ fractions with outcome after therapy, although these populations may contain tumor-reactive cells[35,38]. In contrast to the latter, little is known about the heterogeneous CD56+ T-cell populations and their PD-1-positivity in human cancer. CD56+ T cells consist mainly of type I[15,16] and II[17,18] NKT-cells and certain other, non-MHC-restricted T-cells such as γδ T-cells[20,22], and are commonly described as immune invigorating cells including those producing T helper 1 cytokines and mediating efficient cytotoxicity[39]. The latter might be impaired in cancer: frequencies of CD56+ T-cells in AML and ALL patients achieving remission on chemotherapy returned to those in healthy controls, although their functionality was still impaired[24]. Interestingly Achberger *et*. *al* found changes in the phenotypic composition of circulating CD56+ T-cells in primary uveal melanoma. Development of metastasis was associated with a decrease of peripheral DN and CD8+CD56+ T-cells, whereas the frequency of CD4+CD56+ T-cells was not altered, suggesting a potential involvement of DN and CD8+CD56+ T-cells in cancer immunosurveillance in uveal melanoma[40]. CD4 and/or CD8 is differently expressed on type I or type II NKT-cells[15–18] or even on γδ T-cells[21] and might be used to conclude associations between identified phenotypes and functional capabilities. However, the here identified relevance of PD-1+CD56+ T-cells prior to the initiation of PD-1 blockade was not linked with the presence/absence of CD4 and/or CD8 as visualized in an unbiased tSNE analysis approach. Further investigation on the basis of this approach was not performed due to limitations of the algorithm when run on polychromatic flow cytometry data.

Tumor infiltration by CD56+ T-cells has been associated with cancer rejection[39,41,42]. For example, high tumor infiltration rates of CD56+ T-cells correlated in gastric cancer with prolonged OS, while their PD-1 expression levels did not differ compared to non-tumor tissue resident cells[43]. But not every CD56+ T-cell acts in an immune stimulatory manner. Immunosuppressive capabilities of these cells in the tumor microenvironment have also been reported[22,44]. However, the here revealed negative associations between high peripheral PD-1+CD56+ T-cell frequencies and the course of disease does not allow a final conclusion as to whether the mechanistic contribution of these cells in the ensemble of immune-mediated cancer rejection under anti-PD-1 immune therapy is positive or negative. This functional analysis remains to be performed in future studies.

Nonetheless, we considered in this study major potential confounding features that might impact T-cell immunomonitoring to achieve best statistical accuracy[45,46]. Seropositivity for human herpesvirus V (Cytomegalovirus, CMV)[28,33] has a well-documented impact on T cell subset distributions. For the first time, to the best of our knowledge, CMV-IgG seropositivity was considered in a T-cell immunomonitoring study of melanoma patients under immune checkpoint therapy. Subset analysis of our cohort revealed lower CD4+ and higher CD8+ and CD56+ T-cell frequencies in CMV-seropositive than in -seronegative patients, consistent with observations in healthy elderly subjects[28]. However, CMV seropositivity had, despite increasing the heterogeneity within the T-cell subsets, no significant impact on clinical outcome after PD-1 blockade. We also found no correlation of the former with sex or age. Further, a descriptive, stepwise multivariate analysis to determine dependencies between this biomarker candidate and other potentially informative features identified the abundance of PD-1+CD56+ T-cells as the only independent predictor of patients’ OS apart from the M category.

Thus, we have identified a strong biomarker candidate for clinical outcome after PD-1 immune checkpoint therapy based on the frequency of PD-1+CD56+ T-cells in peripheral blood. Confirmation and validation of these findings is required in future studies. Further, comparative investigation of these cells and the PD-1 expression intensity, as recently reported [47], in corresponding tumor tissue and peripheral blood samples of melanoma patients and healthy donors will be warranted to learn more about potential migration patterns, functional and phenotypical characteristics and could help to understand why patients with low PD-1+CD56+ T-cell frequencies have better chances of benefiting from therapy.

## Supporting information

S1 Fig Gating strategy for the flow cytometry-based analysis of CD56+ and CD56- T-cell populations. Here, we display a representative data set from one patient to illustrate the flow cytometry gating strategy. The first plot shows the SSC-A channel versus measured time to monitor unexpected alterations in the pressure system of the LSR II (BD) flow cytometer. Next, debris (SSC-A versus FSC-A) and duplicates (FSC-A versus FSC-H and SSC-A versus SSC-H) were excluded. Viable cells were selected by excluding EMA-positive cells (EMA versus FSC-A). A morphological gate was used to select the lymphocyte population (SSC-A versus FSC-A). The thus selected lymphocyte population was divided into CD56+CD3+ and CD56-CD3+ cells. These T-cells were further separated into CD4+, CD8+, CD4-CD8- and CD4+CD8+ T-cells. Finally, the frequencies of PD-1+ fractions on the selected T-cell subsets were determined.
(PDF)

S2 Fig Impact of CMV seropositivity on the observed peripheral T-cell compartment. The frequency of CD56- and CD56+ T-cell populations (A) and the frequencies of the PD-1+ fraction within these populations (B) are compared between CMV seronegative (CMV-, n = 21) and seropositive (CMV+, n = 53) melanoma patients. Horizontal lines in each plot show the median and each symbol represents an individual patient; * p ≤ 0.05, ** p ≤ 0.01, *** p < 0.001, using the Mann-Whitney-U-test.
(PDF)

S3 Fig Correlation of the peripheral PD-1+CD56+ T-cell subset with progression-free survival (PFS). Stratification of the patient cohort according to PD-1+CD56+ T-cell frequencies (≤16.6% [green]; >16.6% [blue] PD-1+CD56+ T-cells) reveals a significant correlation of the frequencies of these cells with PFS (p = 0.041, log-rank test) using the Kaplan-Meier method. Vertical lines indicate censored events.
(PDF)

S4 Fig Univariate analysis of correlations between variables and OS. Stratification of the cohort according to the following features: PD-1+CD4+ T-cells, PD-1+CD8+ T-cells, serum LDH, M-category, sex, age and CMV-serostatus for associations with patient survival using the Kaplan-Meier method. Vertical lines indicate censored events and p-values were estimated by log-rank testing.
(PDF)

S1 Table Anonymized raw dataset. (CSV)
