# Supplementary figures and images for: Peripheral PD-1+CD56+ T-cell frequencies correlate with outcome in stage IV melanoma under PD-1 blockade
Source: PLoS One. 2019 Aug 16;14(8):e0221301. doi: 10.1371/journal.pone.0221301 (PMC6697319; doi:10.1371/journal.pone.0221301)

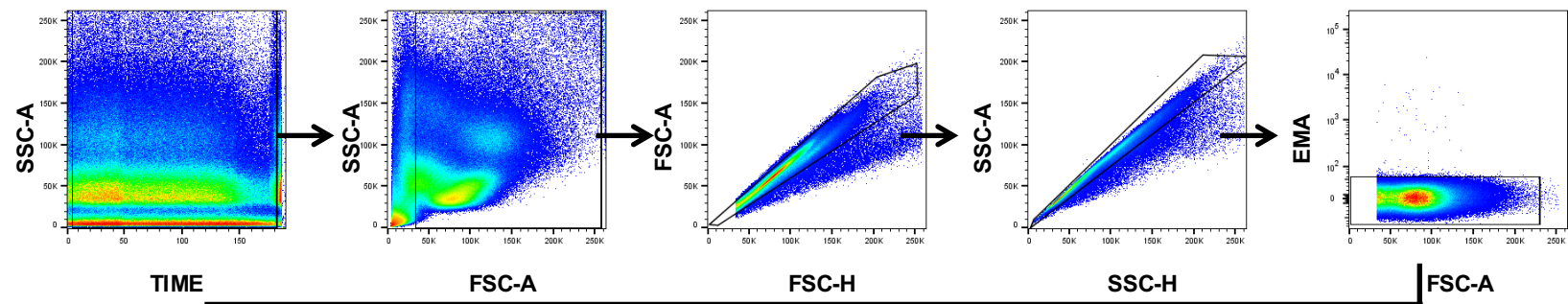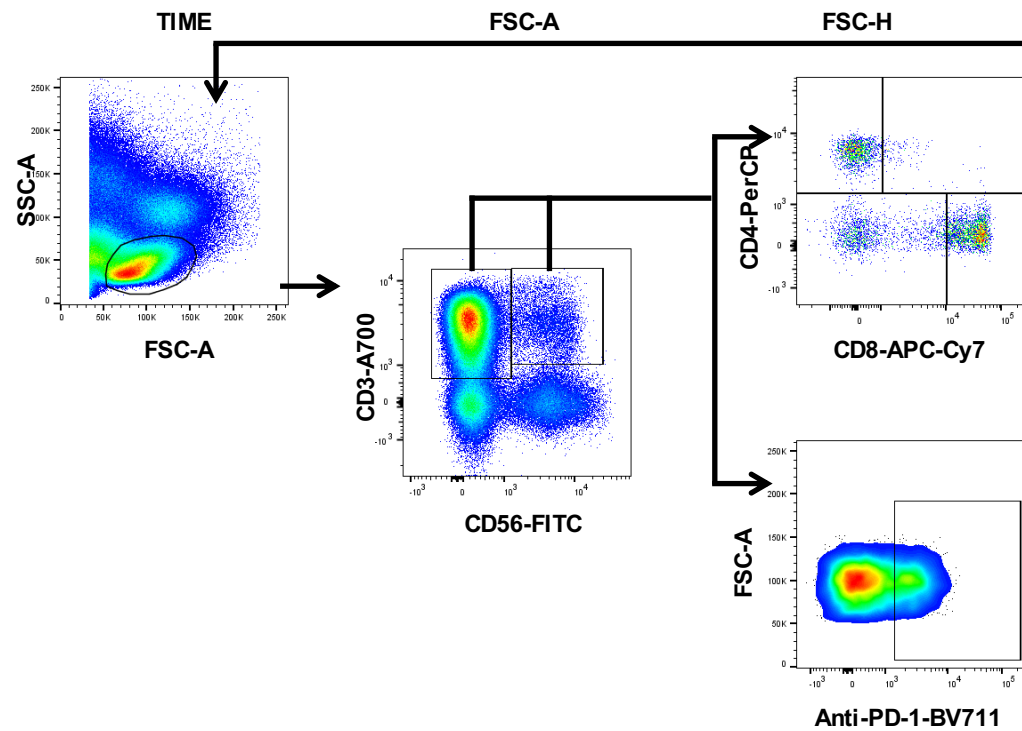

Supplement: S1 Fig — Here, we display a representative data set from one patient to illustrate the flow cytometry gating strategy. The first plot shows the SSC-A channel versus measured time to monitor unexpected alterations in the pressure system of the LSR II (BD) flow cytometer. Next, debris (SSC-A versus FSC-A) and duplicates (FSC-A versus FSC-H and SSC-A versus SSC-H) were excluded. Viable cells were selected by excluding EMA-positive cells (EMA versus FSC-A). A morphological gate was used to select the lymphocyte population (SSC-A versus FSC-A). The thus selected lymphocyte population was divided into CD56+CD3+ and CD56-CD3+ cells. These T-cells were further separated into CD4+, CD8+, CD4-CD8- and CD4+CD8+ T-cells. Finally, the frequencies of PD-1+ fractions on the selected T-cell subsets were determined. (PDF) [file pone.0221301.s001.pdf]

**A**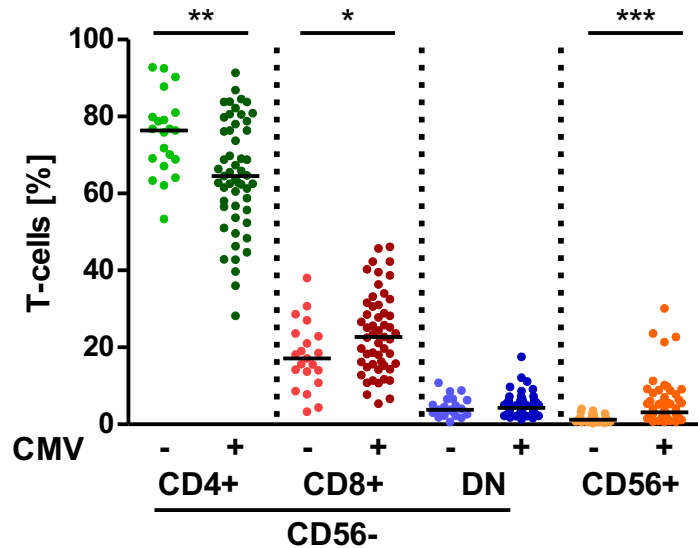**B**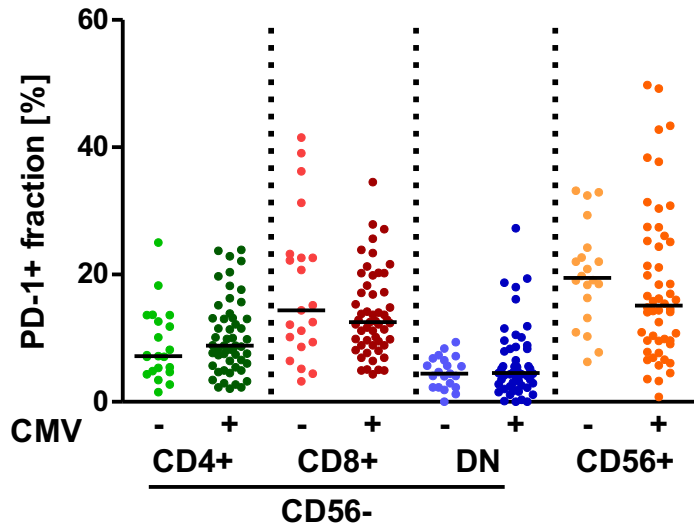

Supplement: S2 Fig — The frequency of CD56- and CD56+ T-cell populations (A) and the frequencies of the PD-1+ fraction within these populations (B) are compared between CMV seronegative (CMV-, n = 21) and seropositive (CMV+, n = 53) melanoma patients. Horizontal lines in each plot show the median and each symbol represents an individual patient; * p ≤ 0.05, ** p ≤ 0.01, *** p < 0.001, using the Mann-Whitney-U-test. (PDF) [file pone.0221301.s002.pdf]

## Progression-free Survival

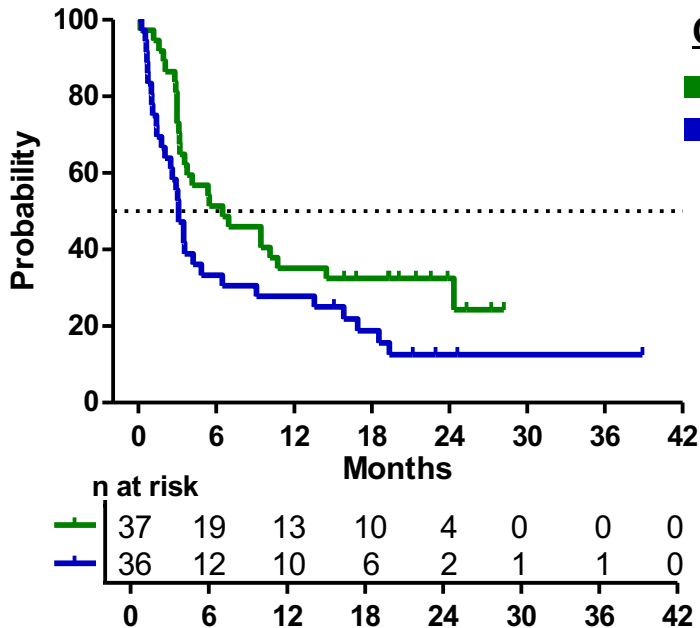

Supplement: S3 Fig — Stratification of the patient cohort according to PD-1+CD56+ T-cell frequencies (≤16.6% [green]; >16.6% [blue] PD-1+CD56+ T-cells) reveals a significant correlation of the frequencies of these cells with PFS (p = 0.041, log-rank test) using the Kaplan-Meier method. Vertical lines indicate censored events. (PDF) [file pone.0221301.s003.pdf]

**PD-1+CD4+ T-cells**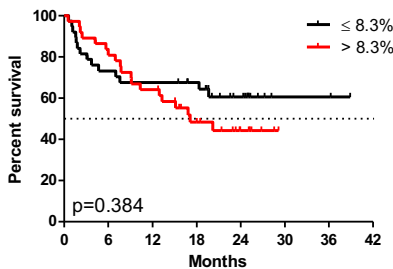**PD-1+CD8+ T-cells**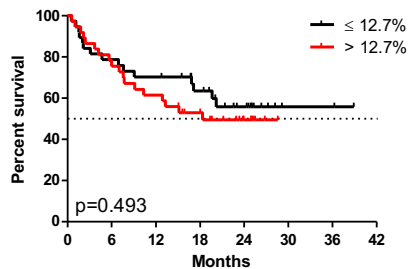**LDH**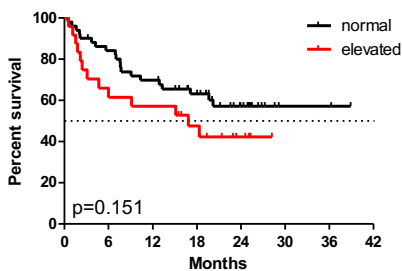**M-category**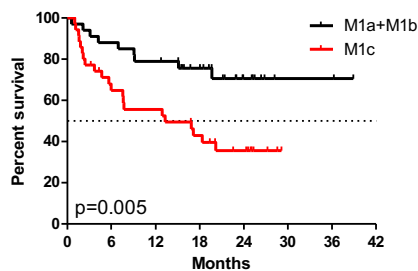**Age**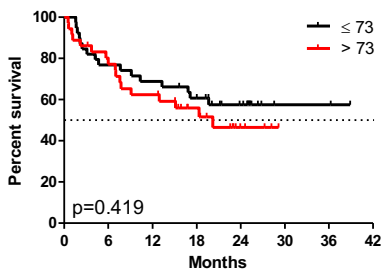**Sex**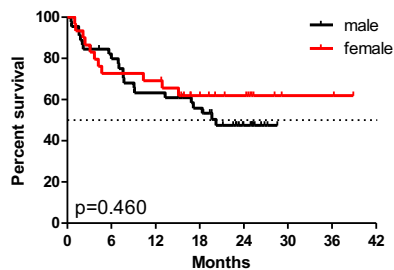**CMV-serostatus**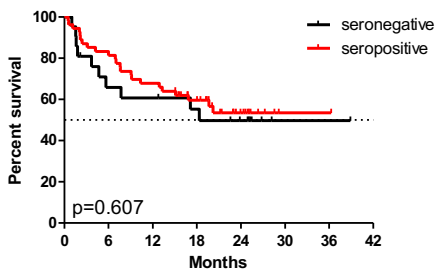

Supplement: S4 Fig — Stratification of the cohort according to the following features: PD-1+CD4+ T-cells, PD-1+CD8+ T-cells, serum LDH, M-category, sex, age and CMV-serostatus for associations with patient survival using the Kaplan-Meier method. Vertical lines indicate censored events and p-values were estimated by log-rank testing. (PDF) [file pone.0221301.s004.pdf]
